# Global burden of esophageal cancer attributable to high BMI in 204 countries and territories: 1990–2019

**DOI:** 10.1111/1759-7714.15239

**Published:** 2024-02-05

**Authors:** Zhiming Chen, Xingxing Zhang, Jianxue Zhai, Jiayang Fan, Yikuan Cai, Tianlan Ye, Zhizhi Wang, Kaican Cai

**Affiliations:** ^1^ Department of Thoracic Surgery, Nanfang Hospital Southern Medical University Guangzhou China; ^2^ Department of General Surgery, Nanfang Hospital Southern Medical University Guangzhou China

**Keywords:** esophageal cancer, global burden of disease (GBD), high body mass index (BMI)

## Abstract

**Background:**

Esophageal cancer (EC), a common and fatal disease, includes two histological subtypes; esophageal squamous cell carcinoma (ESCC) and esophageal adenocarcinoma (ECA). To aid policymakers in the allocation of resources for the prevention and treatment of EC, updated data on EC deaths and disability‐adjusted life years (DALYs) attributable to high body mass index (BMI) are necessary. The objective of this study was to identify trends in EC associated with high BMI between 1990 and 2019 using 2019 Global Burden of Disease data.

**Methods:**

In this observational population‐based study, epidemiological data on the association between high BMI and EC were obtained from GBD 2019. The age‐standardized mortality rate (ASMRs) and disability‐adjusted life year rate (ASDRs) attributable to high BMI‐related EC were stratified by year, age, country, and sociodemographic index (SDI). The estimated annual percentage change (EAPC) was calculated to evaluate the temporal trends of the ASMRs and ASDRs between 1990 and 2019.

**Results:**

In 2019, the proportion of EC deaths and DALYs attributed to high BMI was 18.1% and 18.9%, respectively, resulting in 89 904 (95% confidence interval [CI]: 27 879–171 255) deaths and 2 202 314 (95% CI: 681 901–4 173 080) DALYs. High BMI‐related deaths and DALYs showed a strong upward trend, increasing by more than two‐fold since 1990. East Asia and Western Europe showed the highest risk of EC mortality and DALYs attributable to high BMI; China and the USA bear the greatest burden. The ASMR and ASDR increased in five SDI regions.

**Conclusions:**

The incidence of EC is increasing, particularly in developing nations, which may be attributed to the prevalence of high BMI. To mitigate the impact of high BMI on the incidence of EC, it is important to increase awareness of its deleterious effects, which may alleviate the burden of this disease.

## Graphical abstract

The spatial distribution of esophageal cancer ASMR (A) and ASDR (B) attributable to high BMI in 2019.

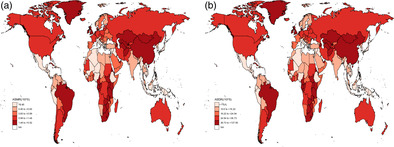

## INTRODUCTION

Esophageal cancer (EC) is a malignancy of the digestive system that exhibits high incidence and mortality rates. In 2020, EC was ranked as the seventh most prevalent cancer (604 000 new cases, 3.1%) and the sixth leading cause of cancer‐related death (544 000 deaths, 5.5%) worldwide.^1^ EC is classified into two histopathological types; esophageal squamous cell carcinoma (ESCC) and esophageal adenocarcinoma (EAC). ESCC is the predominant type globally, accounting for >85% of cases,^2^ whereas EAC is the predominant histological type in some Western countries, and its incidence has recently increased.^3^ More than half of the patients have metastases at the time of diagnosis because of the lack of early symptoms prior to local progression or metastatic deposition.^4^ Despite improvements in early detection and treatment strategies, the 5‐year survival rate of EC remains <20%.^5^ The epidemiology of EC has undergone significant changes because of the increased life expectancy and economic growth.^6^

High body mass index (BMI) is the primary lifestyle‐associated factor related to untimely mortality, and it increases the incidence and fatality of many diseases including cardiovascular disease, type 2 diabetes mellitus, nonalcoholic fatty liver disease, and certain types of cancer.^7^, ^8^ Cancer is defined as a group of illnesses caused by the interplay between genetic and environmental or behavioral factors, and a high BMI ranks third among behaviorally linked risk factors after smoking and infection.^9^ Despite being a modifiable behavioral risk factor, high BMI has not received adequate attention in the realm of oncology, particularly in relation to EC. To effectively address this issue, it is imperative to obtain reliable and timely quantitative data at both regional and global levels. The association between EC and body fat, which is commonly assessed by calculating BMI, has been investigated in numerous epidemiological studies. These investigations have consistently revealed a positive correlation between BMI and the likelihood of developing EAC, whereas a negative correlation has been observed between BMI and the risk of ESCC.^10^, ^11^, ^12^, ^13^, ^14^, ^15^ Previous meta‐analyses identified ethnic disparities in the correlation between BMI and the incidence of EC. Specifically, in European and North American populations, increased BMI is positively associated with the overall risk of EC, whereas in Asian populations, it is inversely associated with the risk.^14^, ^15^ Most of the existing research has been carried out in Western cohorts, which are characterized by a high average BMI and a low prevalence of EC. However, there is a paucity of reliable epidemiological data linking BMI to EC mortality in Asian populations, in which the mean BMI is comparatively low and the incidence of EC is high.^16^

The 2019 Global Burden of Disease (GBD) collaborative group assessed the disease burden caused by high BMI in terms of death and disability‐adjusted life years (DALYs), thereby providing an opportunity to assess the influence of high BMI on EC The objective of this study was to comprehensively evaluate the burden of EC‐related deaths and DALYs associated with high BMI at the global, regional, and national levels using data from GBD 2019. We also assessed the correlation between the EC burden, sociodemographic index (SDI), and human development index (HDI). The estimated annual percentage change (EAPC) was calculated to quantify the secular trends of the age‐standardized rate (ASR). These data and their variations were then used to explore potential directions for primary prevention, screening, early diagnosis, and treatment of EC related to high BMI.

## METHODS

### Data sources

All the data used in this research were derived from GBD 2019, the objective of which is to assess country‐level variability, analyze health trends across time, and develop global disease control strategies.^17^ Data were collected from the Global Health Data Exchange query tool (http://ghdx.healthdata.org/gbd-results-tool). The Institute for Health Metrics and Evaluation (IHME) created and maintains the Global Health Data Exchange, a database that offers a download interface for GBD results data. The search criteria were “esophageal cancer” as the keyword, “high BMI” as the risk factor, “death, DALYs” as the measurements, “1990–2010, 1990–2019, and 2010–2019” as the year ranges, and “number, percent and rate” as the metrics. The United Nations Development Program (http://hdr.undp.org/en/data) provided the HDI statistics.

### Definitions

The definition of high BMI includes individuals aged ≥20 years with a BMI of 25 kg/m^2^ or higher.^18^ The calculation of DALYs involves the summation of years lost due to premature mortality and years lived with disability. The methods used for modeling cause‐specific deaths and DALYs have been described elsewhere.^17^ The sociodemographic index (SDI) serves as a composite indicator that gauges the average per capita income, fertility, and educational level of each region and country. The SDI ranges from 0 to 1, representing the lowest to the highest level of development, respectively. Regions and countries are divided into five groups according to the SDI: high SDI (>0.81), high‐moderate SDI (0.70–0.81), moderate SDI (0.61–0.69), low‐moderate SDI (0.46–0.60), and low SDI (<0.46).

### Statistical analysis

GBD collaborators have reported the inclusion of high BMI data and detailed methods for estimating deaths and DALYs.^19^ Deaths, DALYs, ASMRs, and ASDRs were calculated with the corresponding 95% confidence interval (CI) to quantify the burden of EC attributable to high BMI. The global age structure from GBD 2019 was used to normalize age‐standardized rates, and the population attributable fractions were used to assess the ASMRs and ASDRs related high BMI.^18^ Age standardization of rates eliminates the confounding effect of differences in age structure in the population being compared using the following formula:

$\mathrm{ASR}=\frac{\sum_{i=1}^{A}a_{i}w_{i}}{\sum_{i=1}^{A}w_{i}}\times 100000$

wherein a_i_ denotes the age‐specific rate in the ith age group, w_i_ denotes the number or weight of individuals in the same ith age group from the chosen standard population, and A denotes the total number of age groups.

The trend in age‐standardized rates for EC linked to high BMI between 1990 and 2019 was determined and explained using the following formula: *y* = α + βx + ε, where y = ln age‐standardized rate (ASR), x = calendar year, and ε = error term. EAPC = 100 × (exp (β)‐1) with 95% CI obtained from a linear regression model. The trends were defined as follows: an upward trend in ASR was indicated by EAPC and 95% CI >0, whereas a downward trend was indicated by the EAPC and 95% CI <0. Results that did not fit these definitions indicated that the ASR remained stable over time. To investigate the factors affecting the EAPC, Pearson correlation analysis was performed to examine the relationship between the EAPC, ASRs, and country‐level HDI. Lastly, a hierarchical cluster analysis was performed to classify countries and regions into four categories based on their temporal trends in the ASMR and ASDR: (a) remained stable, (b) minor increase, (c) significant increase, and (d) decrease. Data was analyzed using R program (version 4.2.3).

## RESULTS

### Global trends of EC attributable to high BMI

**FIGURE 1:**
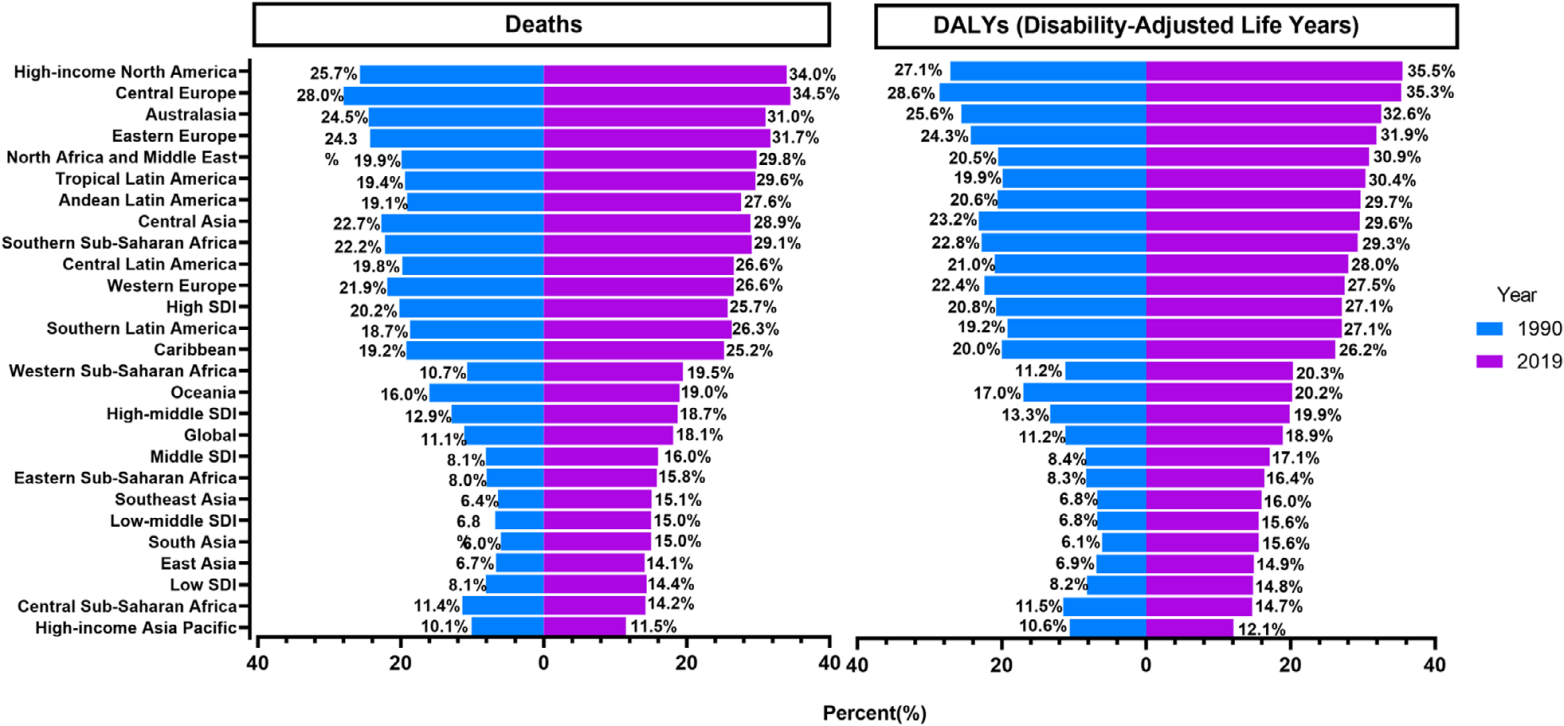
Proportion of esophageal cancer deaths and DALYs attributable to high BMI globally and in 26 GBD regions in 1990 and 2019. DALYs, disability‐adjusted life‐years; GBD, Global Burden of Disease Study.

**FIGURE 2:**
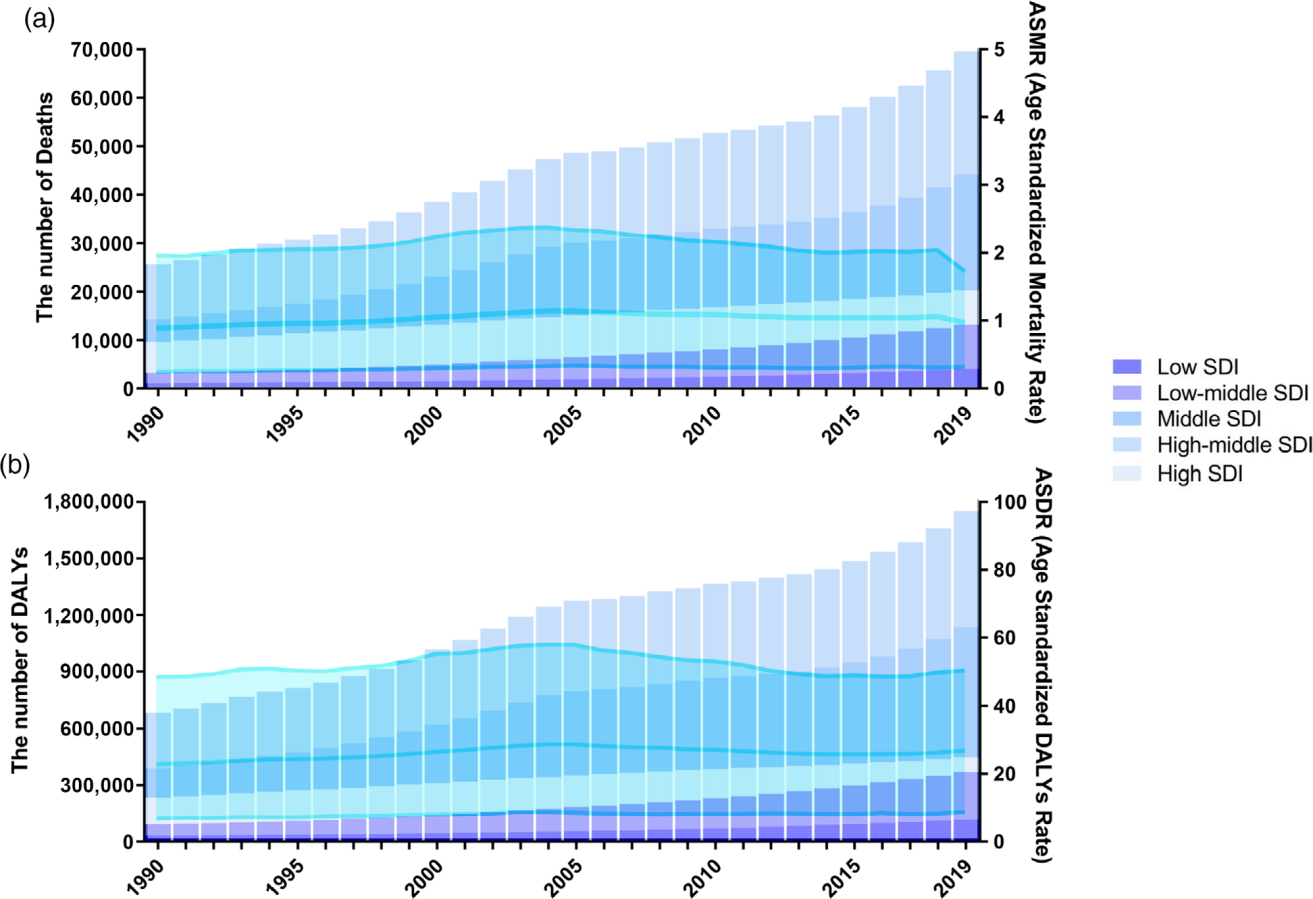
Number and rate of esophageal cancer deaths (a) and DALYs (b) attributable to high BMI between 1990 and 2019 according to the SDI. The bars represent the number of esophageal cancer deaths (a) and DALYs (b) attributable to high BMI colored by SDI level. The line represents the mean ASMR (a) and ASDR (b) (per 100 000) attributable to high BMI at the global level. The shaded area represents the 95% UI for the mean rate. ASMR, age‐standardized mortality rate; ASDR, age‐standardized DALY rate; DALYs, disability‐adjusted life‐years; SDI, socio‐demographic index; UI, uncertainty interval.

Of all EC deaths and DALYs worldwide in 2019, 18.1% and 18.9% were attributable to high BMI, respectively (Figure 1), resulting in 89 904 (95% CI: 27 879–171 255) EC deaths and 2 202 314 (95% CI: 681 901–4 173 080) DALYs due to high BMI in 2019. The global number of EC‐related deaths related to high BMI increased from 35 280 in 1990 to 89 900 in 2019, and the global DALYs for EC due to high BMI increased from 920 000 in 1990 to 2 200 000 in 2019. The ASMR was 1.09 (95% CI: 0.34–2.10) per 100 000 people, and the age‐standardized DALYs rate was 26.27 (95% CI: 8.12–49.89) per 100 000 people in 2019. Time trend analysis showed a significant increase in the ASMR and ASDR for EC attributable to high BMI, with EAPCs of 0.52 (95% CI: 0.30–0.74) and 0.42 (95% CI: 0.19–0.65), respectively (Table 1; Figure 2). The distribution of deaths and DALYs remained stable over time in each of the four age subgroups (File S1).

**TABLE 1:** Global burden of esophageal cancer in 1990 and 2019 for both sexes and all locations, with EAPC.

Characteristics	1990				2019				EAPC(1990–2019)	
	Deaths cases	ASMR per 100 000	DALYs	ASDR per 100 000	Deaths cases	ASMR per 100 000	DALYs	ASDR per 100 000	ASMR	ASDR
	No. × 10^2^ (95% CI)	No. (95% CI)	No. × 10^3^ (95% CI)	No. (95% CI)	No. × 10^2^ (95% CI)	No. (95% CI)	No. × 10^3^ (95% CI)	No. (95% CI)	No. (95% CI)	No. (95% CI)
Global	352.83 (101.82–759.27)	0.9 (0.26–1.97)	917.929 (256.226–1976.144)	22.26 (6.29–47.89)	899.04 (278.79–1712.55)	1.09 (0.34–2.1)	2202.314 (681.901–4173.08)	26.27 (8.12–49.89)	0.52 (0.3–0.74)	0.42 (0.19–0.65)
High SDI	95.8 (29.16–179.18)	0.93 (0.28–1.74)	232.324 (70.647–432.3)	23.26 (7.05–43.3)	202.84 (66.93–355.73)	1.1 (0.36–1.92)	448.011 (145.695–777.638)	26.4 (8.44–45.83)	0.45 (0.32–0.58)	0.31 (0.19–0.44)
High‐middle SDI	113.42 (34.47–237.43)	1.07 (0.33–2.23)	295.329 (82.725–622.129)	26.68 (7.58–55.93)	253.76 (75.8–498.86)	1.23 (0.37–2.42)	616.917 (182.197–1207.438)	29.97 (8.85–58.63)	0.33 (0.16–0.5)	0.22 (0.02–0.42)
Middle SDI	110.82 (24.24–277.89)	1.11 (0.24–2.81)	296.557 (66.679–728.52)	26.94 (6.04–66.87)	310.24 (91.8–626.32)	1.27 (0.38–2.57)	765.974 (224.73–1518.192)	29.29 (8.53–58.12)	0.27 (−0.12–0.66)	0.08 (−0.32–0.49)
Low‐middle SDI	20.87 (5.08–51.1)	0.36 (0.08–0.87)	59.062 (14.438–144.513)	8.98 (2.19–21.99)	90.74 (29.88–178.33)	0.66 (0.22–1.31)	251.342 (82.449–491.135)	17.14 (5.6–33.54)	2.2 (2.1–2.29)	2.32 (2.23–2.4)
Low SDI	11.79 (2.85–27.06)	0.5 (0.12–1.16)	34.313 (8.341–78.611)	12.93 (3.13–29.6)	41.12 (12.01–82.5)	0.79 (0.23–1.58)	119.207 (35.643–239.878)	20.56 (6.06–41.46)	1.69 (1.6–1.78)	1.69 (1.6–1.78)
Andean Latin America	0.78 (0.25–1.53)	0.39 (0.12–0.78)	2.029 (0.653–3.879)	9.44 (3–18.07)	2.45 (0.82–4.49)	0.45 (0.15–0.82)	5.6 (1.897–10.225)	9.87 (3.32–17.96)	0.5 (0.42–0.59)	0.19 (0.1–0.28)
Australasia	2.49 (0.83–4.49)	1.06 (0.35–1.92)	5.69 (1.85–10.182)	24.63 (8–43.92)	6.31 (2.24–10.9)	1.27 (0.45–2.17)	13.021 (4.529–22.378)	28.16 (9.73–48)	0.5 (0.38–0.61)	0.39 (0.29–0.49)
Caribbean	1.96 (0.66–3.7)	0.77 (0.25–1.45)	4.853 (1.611–9.069)	18.42 (6.12–34.44)	4.84 (1.5–8.77)	0.93 (0.29–1.69)	12.409 (3.818–22.637)	23.73 (7.3–43.34)	0.95 (0.7–1.2)	1.21 (0.97–1.45)
Central Asia	15.04 (4.88–27.09)	3.26 (1.03–5.87)	39.632 (13.103–70.992)	81.14 (26.5–145.63)	14.24 (4.89–24.8)	1.99 (0.68–3.45)	38.393 (12.709–67.193)	47.83 (16.43–83.25)	−2.04 (−2.36–1.72)	−2.19 (−2.51–1.86)
Central Europe	12.13 (4.07–21.26)	0.82 (0.28–1.45)	33.036 (10.597–57.504)	22.23 (7.13–38.72)	20.22 (6.9–34.56)	0.99 (0.34–1.69)	50.676 (16.622–87.404)	26.35 (8.34–45.56)	0.5 (0.39–0.61)	0.39 (0.25–0.53)
Central Latin America	3.98 (1.3–7.52)	0.5 (0.16–0.95)	10.108 (3.361–19.012)	11.54 (3.78–21.82)	10.69 (3.64–19.49)	0.46 (0.16–0.84)	25.414 (8.466–46.217)	10.55 (3.53–19.18)	−0.51 (−0.6–0.42)	−0.5 (−0.59–0.41)
Central sub‐Saharan Africa	2.86 (0.71–6.54)	1.25 (0.3–2.88)	8.29 (2.05–19.019)	32.17 (7.91–73.85)	6.39 (1.71–13.49)	1.2 (0.32–2.61)	18.649 (4.948–38.47)	30.62 (8.21–64.41)	−0.76 (−1.15–0.36)	−0.77 (−1.16–0.38)
East Asia	119.87 (19.84–326.89)	1.41 (0.23–3.88)	312.386 (50.58–839.095)	33.51 (5.5–91.09)	369.4 (96.21–814.95)	1.77 (0.46–3.9)	881.413 (224.528–1929.647)	40.4 (10.38–88.17)	0.66 (0.19–1.14)	0.51 (0–1.02)
Eastern Europe	29.55 (9.78–54.09)	1.04 (0.36–1.89)	78.159 (25.458–143.685)	27.21 (8.88–50.12)	33.81 (11.41–59.42)	0.98 (0.33–1.73)	88.533 (28.263–159.064)	26.52 (8.41–47.25)	−0.54 (−0.8–0.29)	−0.46 (−0.73–0.18)
Eastern sub‐Saharan Africa	6.91 (1.49–16.68)	0.91 (0.19–2.23)	20.354 (4.561–48.846)	24.06 (5.27–57.93)	26.73 (8.03–54.84)	1.63 (0.47–3.34)	77.843 (23.372–157.367)	42.18 (12.69–85.8)	2.24 (2.14–2.35)	2.16 (2.06–2.25)
High‐income Asia Pacific	10.26 (2.11–24.55)	0.51 (0.11–1.21)	25.779 (4.992–61.948)	12.35 (2.49–29.76)	18.74 (3.77–42.22)	0.42 (0.08–0.95)	37.045 (7.142–82.943)	9.47 (1.89–21.13)	−0.99 (−1.14–0.83)	−1.33 (−1.51–1.14)
High‐income North America	32.22 (10.5–58.08)	0.95 (0.31–1.71)	79.194 (26.082–142.707)	24.42 (8.02–43.78)	81.99 (27.75–135.07)	1.31 (0.45–2.16)	186.23 (62.5–304.731)	31.54 (10.62–51.67)	1.04 (0.83–1.24)	0.77 (0.58–0.96)
North Africa and Middle East	8.8 (2.6–16.44)	0.51 (0.15–0.96)	25.027 (7.516–47.286)	13.16 (3.91–24.7)	29.62 (10.3–50.41)	0.71 (0.25–1.21)	79.894 (27.248–136.935)	16.82 (5.78–28.62)	1.1 (1.03–1.16)	0.8 (0.73–0.88)
Oceania	0.1 (0.03–0.21)	0.34 (0.1–0.73)	0.31 (0.093–0.653)	8.9 (2.6–18.62)	0.28 (0.08–0.56)	0.4 (0.12–0.83)	0.848 (0.253–1.684)	10.2 (3.01–20.44)	0.39 (0.25–0.53)	0.31 (0.13–0.49)
South Asia	15.39 (3.61–37.9)	0.28 (0.06–0.68)	45.229 (10.677–109.505)	7.05 (1.66–17.26)	81.16 (25.29–160.15)	0.57 (0.18–1.13)	230.148 (73.617–449.398)	15.13 (4.81–29.65)	2.42 (2.2–2.65)	2.59 (2.41–2.78)
Southeast Asia	4.57 (1.04–11.17)	0.17 (0.04–0.43)	13.642 (3.19–32.852)	4.7 (1.09–11.42)	23.09 (7.28–45.64)	0.37 (0.12–0.75)	64.696 (20.148–126.435)	9.61 (2.98–18.79)	2.63 (2.56–2.7)	2.46 (2.38–2.54)
Southern Latin America	6.62 (2.06–12.86)	1.46 (0.45–2.85)	15.42 (4.576–30.335)	33.07 (9.72–65.02)	10.71 (3.66–19.06)	1.27 (0.44–2.25)	22.526 (7.763–40.271)	27.42 (9.41–49.06)	−0.92 (−1.17–0.66)	−1.09 (−1.33–0.85)
Southern sub‐Saharan Africa	8.33 (2.67–15.65)	3.01 (0.94–5.65)	24.375 (7.785–45.483)	80.79 (26.09–152.28)	17.76 (6.33–30.23)	3.27 (1.15–5.51)	46.78 (17.149–79.226)	78.16 (28.57–132.81)	−0.14 (−0.69–0.42)	−0.61 (−1.18–0.04)
Tropical Latin America	12.03 (3.57–22.68)	1.31 (0.41–2.49)	33.877 (10.047–63.913)	34.1 (10.13–64.18)	37.85 (12.77–65.16)	1.55 (0.52–2.66)	100.018 (33.039–172.467)	39.75 (13.2–68.52)	0.66 (0.52–0.81)	0.62 (0.46–0.78)
Western Europe	56.9 (17.88–104.35)	1.01 (0.31–1.86)	134.831 (41.681–250.504)	25.28 (7.77–47.21)	92.79 (30.01–163.33)	1.05 (0.34–1.84)	194.261 (62.758–342.685)	24.6 (7.81–43.61)	−0.02 (−0.14–0.1)	−0.27 (−0.4–0.14)
Western sub‐Saharan Africa	2.02 (0.56–4.43)	0.23 (0.06–0.5)	5.71 (1.613–12.222)	5.95 (1.67–12.86)	9.97 (3.16–18.94)	0.54 (0.17–1.02)	27.917 (8.772–53.122)	13.37 (4.24–25.51)	3.28 (3.15–3.41)	3.09 (2.96–3.22)

Abbreviations: ASMR, age‐standardized mortality rate; ASDR, age‐standardized DALY rate; CI, confidence interval; DALYs, disability‐adjusted life‐years; EAPC, estimated annual percentage change; No, number; SDI, sociodemographic index.

### Geographical variations in EC attributable to high BMI

For SDI regions, the middle SDI region had the highest number of high BMI‐related EC deaths (31 024 [95% CI: 9180–62 632]) and DALYs (765 974 [95% CI: 224 730–1 518 192]) in 2019, both accounting for >30% globally. The ASMR and ASDR increased in all five SDI regions between 1990 and 2019. Among these, the low‐middle and low SDI regions experienced faster growth than the high, high‐middle, and middle SDI regions (Table 1).

In terms of geographic region, the highest burden of EC attributable to high BMI was identified in East Asia with 36 940 (95% CI: 9621–81 495) deaths and 881 413 (95% CI: 224 528–1 929 647) DALYs, followed by Western Europe with 9279 (95% CI: 3001–16 333) deaths and 194 261 (95% CI: 62 758–342 685) DALYs, and high‐income North America with 8199 (95% CI: 2775–13 507) deaths and 186 230 (95% CI: 62 500–304 731) DALYs. There were significant variations in the ASMR and ASDR for EC attributable to high BMI. The highest ASMR and ASDR were observed in Southern sub‐Saharan Africa (3.27 deaths and 78.16 DALYs per 100 000), whereas the lowest ASMR and ASDR were observed in Southeast Asia (0.37 deaths and 9.61 DALYs per 100 000). Between 1990 and 2019, the largest decreases in ASMR and ASDR were observed in Central Asia, with EAPCs of −2.04 (95% CI: −2.36, −1.72) and −2.19 (95% CI: −2.51, −1.86), whereas the largest increases were observed in Western sub‐Saharan, with EAPCs of 3.28 (95% CI: 3.15–3.41) and 3.09 (95% CI: 2.96–3.22), respectively (Table 1).

In 1990, there was a four‐fold difference between the regions with the highest and lowest percentage of EC deaths and DALYs associated with high BMI. The regions with the highest percentages were Central and Eastern Europe, high‐income North America, and Australasia, whereas the regions with the lowest percentages were South and Southeast and East Asia. In 2019, the distribution of EC attributable to high BMI exhibited a similar regional pattern, with the highest percentages observed in high‐income North America, Central Europe, and Australasia, and the lowest percentages in high‐income Asia Pacific, Central sub‐Saharan Africa, and East Asia. The contribution of high BMI to the total number of deaths and DALYs due to EC increased in all GBD regions between 1990 and 2019 (Figure 1).

### Country‐level burden of EC attributable to high BMI

**FIGURE 3:**
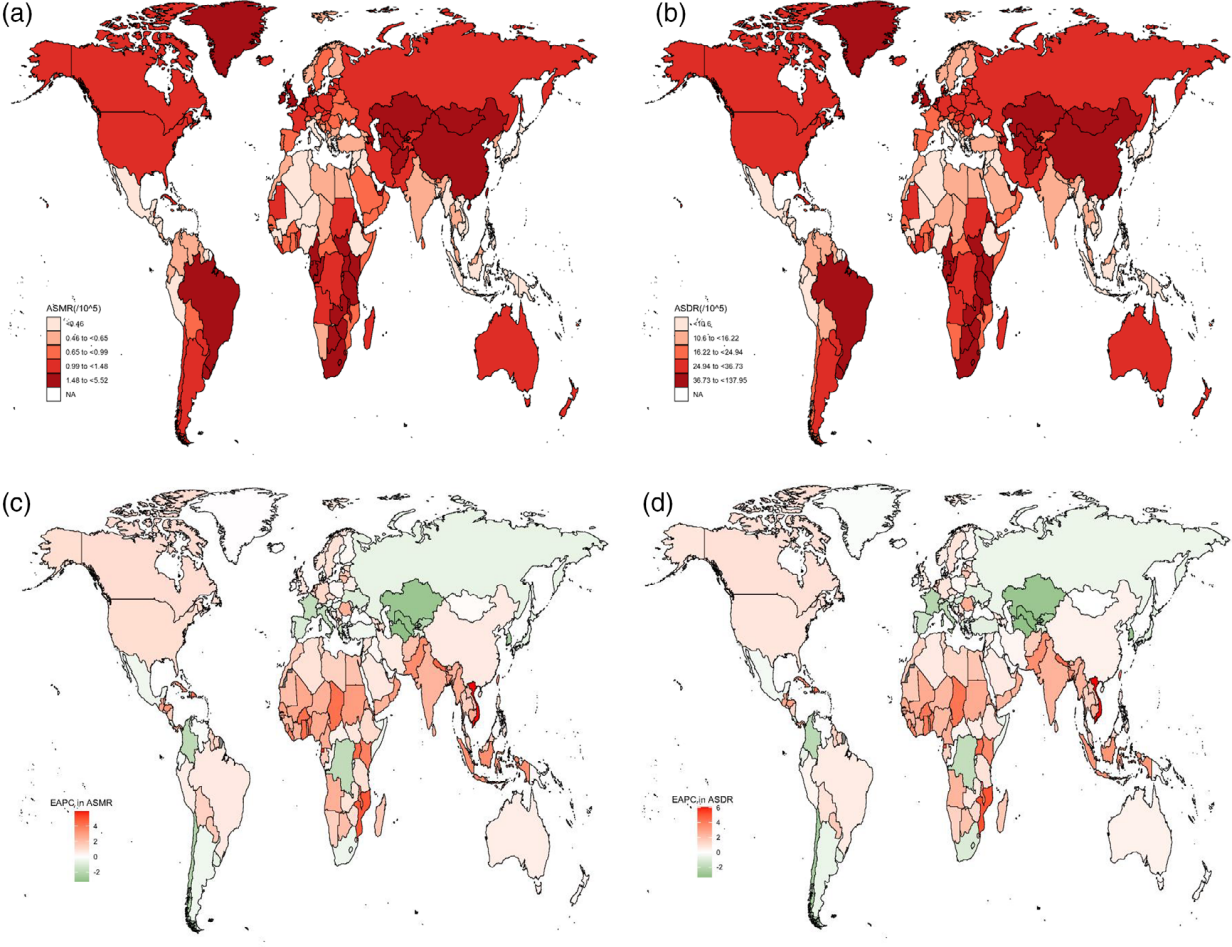
The spatial distribution of the esophageal cancer ASMR (a) and ASDR (b) attributable to high BMI in 2019, and the EAPC in esophageal cancer ASMR (c) and ASDR (d) attributable to high BMI. ASMR, age‐standardized mortality rate; ASDR, age‐standardized DALY rate; EAPC, estimated annual percentage change.

At the country level, China and the USA were the two leading countries for EC‐related deaths due to high BMI, with 36 181 (95% CI: 9426–79 606) and 7428 (95% CI: 2540–12 221) deaths in 2019, respectively. Next were India with 5895 (95% CI: 1788–11 558), Brazil with 3727 (95% CI: 1254–6419), and the UK with 2932 (95% CI: 996–5023). The DALYs followed a similar pattern, with China (859 654 [95% CI: 221 497–1 875 382]), the USA (169 400 [95% CI: 56319–276 895]), and India (165 616 [95% CI: 50 780‐325 517]) as the three leading countries (File S2: Table S1 and S2). Mongolia, Eswatini, and Greenland were the top three countries with the highest EC ASMR attributable to high BMI in 2019; and Eswatini, Mongolia, and Malawi were the top three countries with the highest EC ASDR attributable to high BMI in 2019 (Figure 3a,b; File S2: Tables S3 and S4). The three countries with the highest rates of increase in the ASMR were Vietnam, Equatorial Guinea, and Mozambique, with EAPCs of 5.94 (95% CI: 5.56–6.32), 5.69 (95% CI: 5.27–6.11), and 4.82 (95% CI: 4.56–5.08), respectively; and the three countries with the highest rates of increase in ASDR following the pattern of ASMR were Vietnam, Equatorial Guinea, and Mozambique, with EAPCs of 6.07 (95% CI: 5.65–6.49), 5.06 (95% CI: 4.66–5.46), and 5.04 (95% CI: 4.77–5.32), respectively. The most rapid decrease in the ASMR occurred in Turkmenistan, with an EAPC of −3.21 (95% CI: −3.99, −2.43), and the most rapid decrease in the ASDR occurred in Uzbekistan, with an EAPC of −3.37 (95% CI: −3.87, −2.86) (Figure 3c,d; File S2: Table S5 and S6). The percentage change in the fraction of all EC deaths and DALYs attributable to high BMI is shown in Files S3 and S4.

The cluster analysis showed that 111 countries (or territories) were categorized under the “remained stable” group, which included prominent nations such as China, Australia, and New Zealand. Additionally, 55 countries (or territories) were classified under the “minor increase” group, which comprised countries such as Tunisia, Thailand, and Grenada. Furthermore, 26 countries (or territories) were grouped under the “significant increase” category, which included nations such as Vietnam and the Dominican Republic. The remaining 26 countries (or territories) were classified under the “decrease” group, which included countries such as Singapore, Republic of Korea, Turkmenistan, and France (File S5).

### Global EC burden attributable to high BMI by age

**FIGURE 4:**
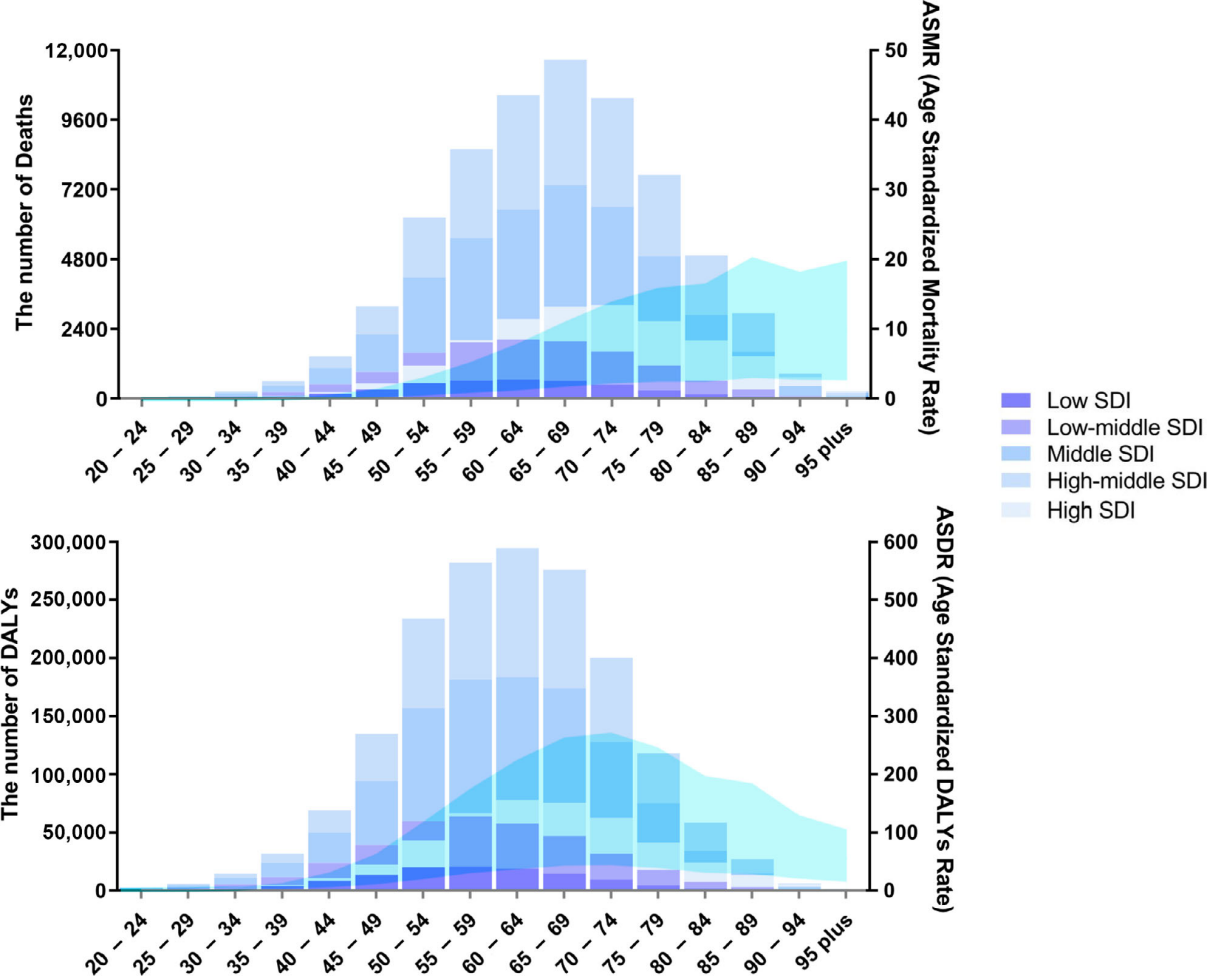
Number and rate of esophageal cancer deaths (a) and DALYs (b) attributable to high BMI by age group and SDI level in 2019. The bars represent the number of esophageal cancer deaths (a) and DALYs (b) attributable to high BMI colored by SDI level. The line represents the mean ASMR (a) and ASDR (b) (per 100 000) attributable to high BMI at the global level. The shaded area represents the 95% UI for the mean rate. ASDR, age‐standardized DALY rate; DALYs, disability‐adjusted life‐years; SDI, socio‐demographic index; UI, uncertainty interval.

In 2019, there was a notable trend in the incidence of deaths from EC linked to high BMI, whereby rates initially increased and subsequently decreased with age. The majority of deaths were observed in the 55–79 age bracket, with a peak in the 65–69 age group. There were more age‐specific deaths in the high‐middle and middle SDI regions than in the low, high, and low‐middle regions, as illustrated in Figure 4a. ASMRs consistently increased from age 20 to 95 years. The pattern of DALYs mirrored that of deaths, with the highest burden of disease occurring in the 55–79 age group and peaking in the 60–64 age group. By contrast, the rate of age‐standardized DALYs gradually decreased after reaching a maximum in the 70–74 age group (Figure 4b).

**FIGURE 5:**
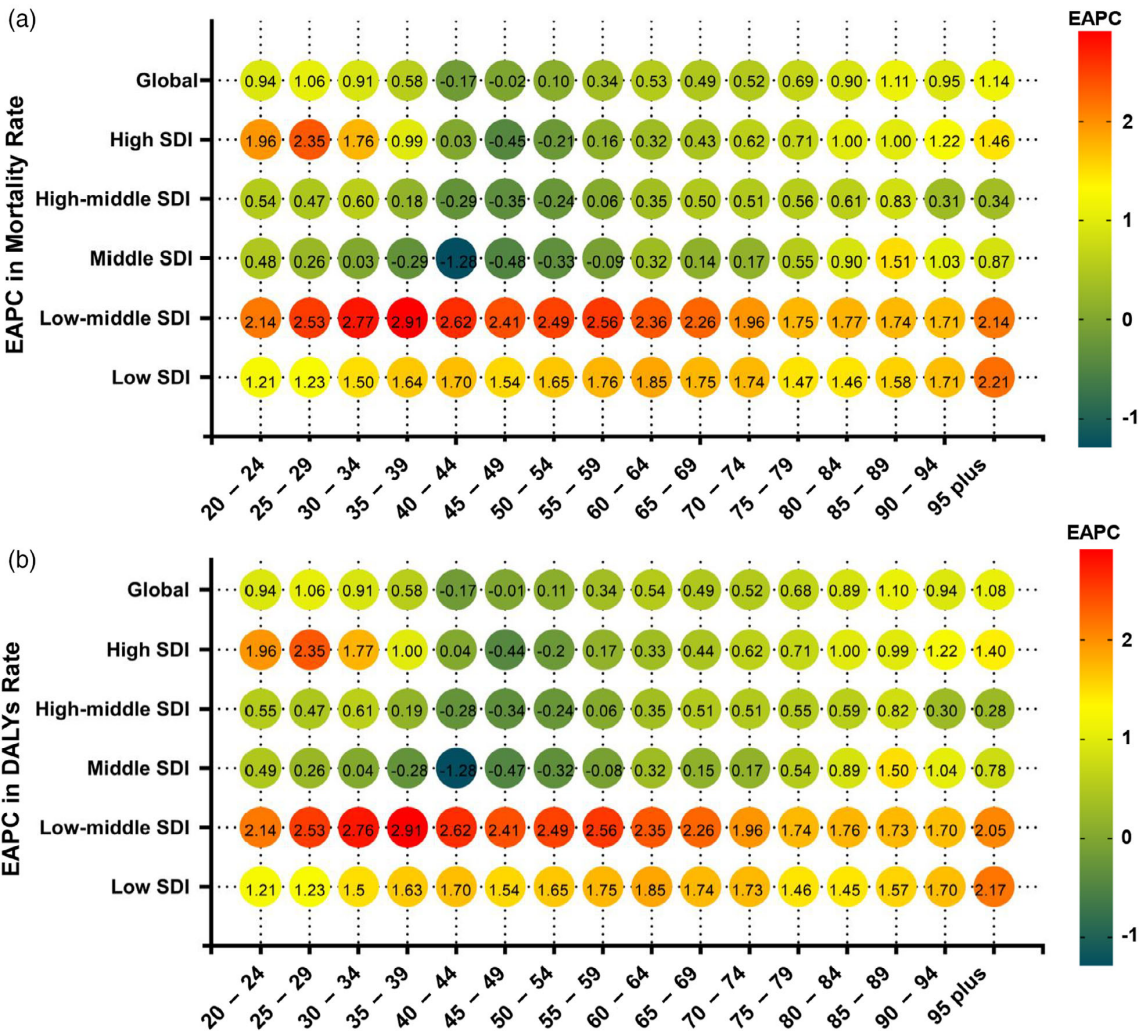
Annual percentage change in mortality (a) and DALYs (b) between 1990 and 2019 by age group and region. EAPC, estimated annual percentage change; SDI, socio‐demographic index; DALYs, disability‐adjusted life‐years.

Globally, age‐standardized mortality rates increased between 1990 and 2019 in almost all age groups, with the fastest growth occurring in the 85–89 age group. The ASMR increased between 1990 and 2019 in low, low‐middle, middle, high‐middle, and high SDI regions; low and low‐middle SDI regions had higher EAPCs for mortality than high‐middle and high SDI regions. In high SDI regions, the ASMR decreased in the 45–54 age group; in high‐middle SDI regions, the ASMR decreased in the 40–54 age group; and in middle SDI regions, the ASMR decreased in the 35–59 age group (Figure 5a). EAPCs in age‐standardized DALY rates showed the same pattern as the ASMR (Figure 5b).

### Factors associated with EC burden attributable to high BMI

**FIGURE 6:**
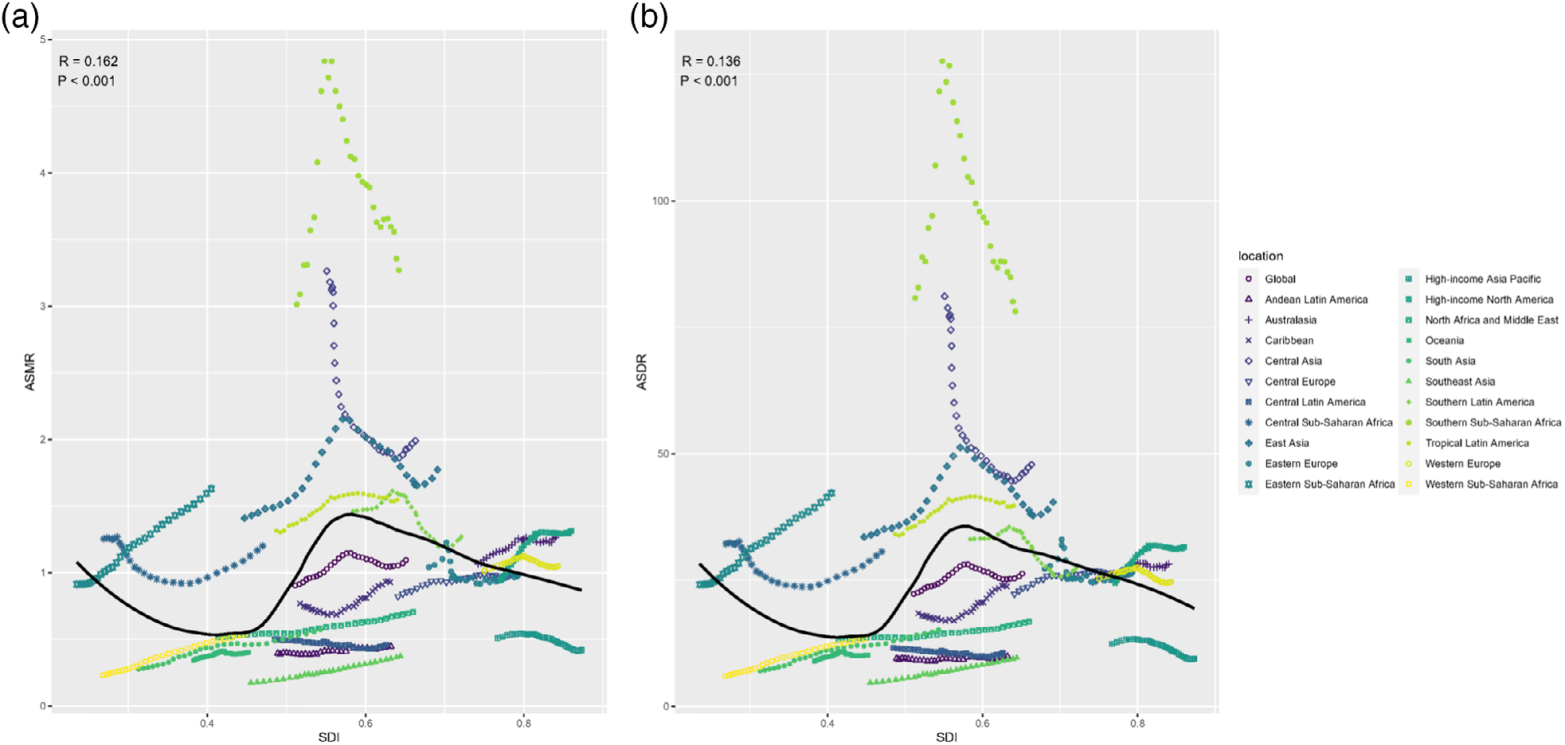
Correlation between high BMI‐attributable esophageal cancer in ASMR or ASDR and SDI globally in 21 GBD regions between 1990 and 2019. ASMR, age‐standardized mortality rate; ASDR, age‐standardized DALY rate; GBD, global burden of disease study.

**FIGURE 7:**
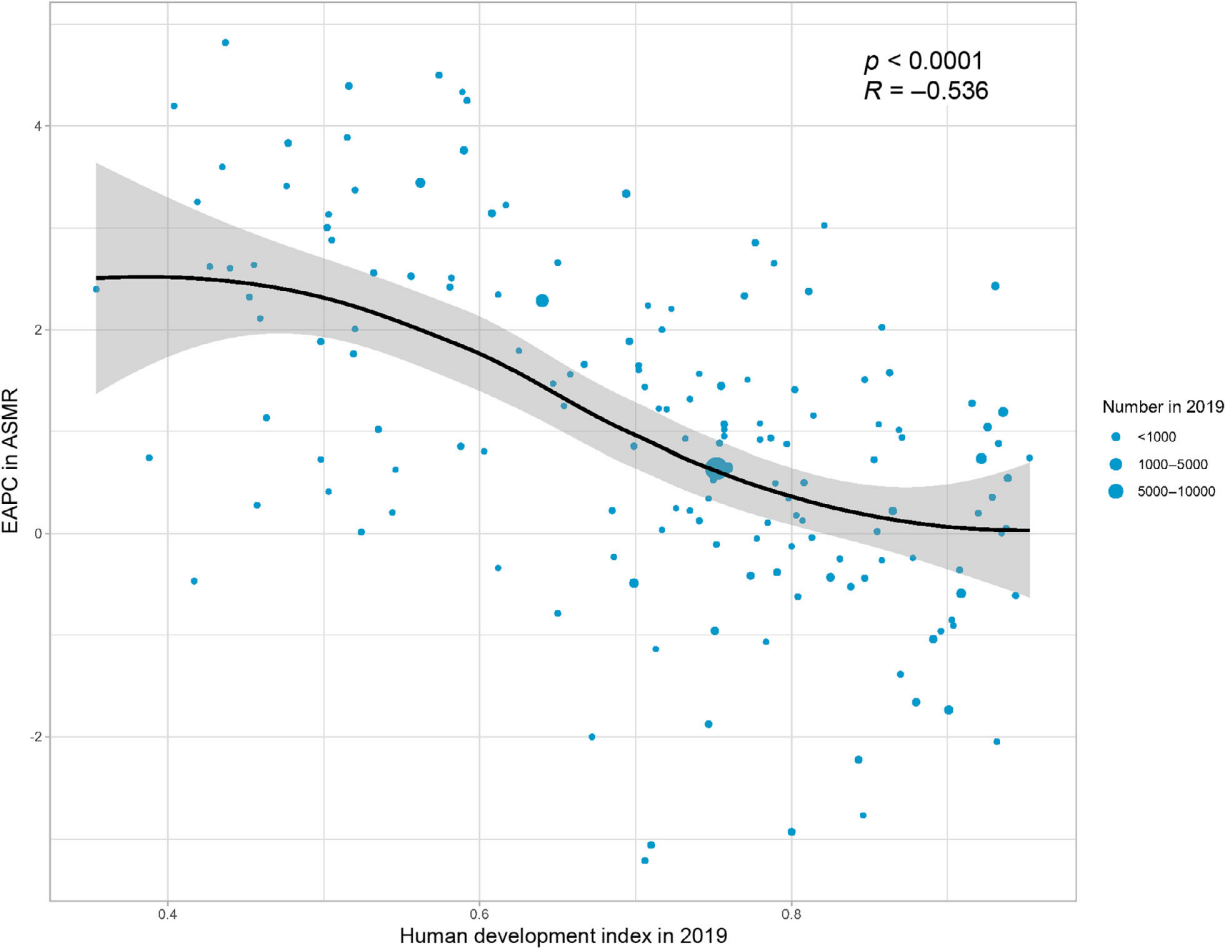
Correlation between EAPC in ASMR and HDI in 2019. ASMR, age‐standardized mortality rate; HDI, human development index.

In 2019, an S‐shaped association between the overall ASMR and ASDR and SDI was observed (Figure 6), with inflection points around 0.45 and 0.55 (Figure 6). The association of the ASMR and ASDR with SDI was visualized for each country. Based on SDI alone, Mongolia, Cabo Verde, Greenland, and Malawi had higher than expected ASMRs or ASDRs (Files S6 and S7). In 2019, there was a significant negative association between the EAPC in the ASMR and HDI (*R* = −0.54, *p* < 0.001) (Figure 7). A highly negative correlation was observed between the EAPC in the ASMR and ASMR in 1990 across different countries (*R* = −0.39, *p* < 0.001) (File S9). The same pattern was observed between the EAPC in the ASDR and HDI in 2019 (*R* = −0.54, *p* < 0.001), and ASDR in 1990 (*R* = −0.38, *p* < 0.001) (Files S8 and S10).

## DISCUSSION

This study summarized the epidemiological characteristics of the global burden of EC associated with high BMI using the latest GBD 2019 data. The findings show that high BMI is responsible for 18.1% of all EC deaths and 18.9% of DALYs. Over the past 30 years, the global trend in EC associated with high BMI has increased slightly in the ASMR and ASDR, but the corresponding absolute number of EC deaths and DALYs has more than doubled globally. The observed rise in global EC‐related mortality and DALYs associated with high BMI can be attributed, in part, to the escalating prevalence of high BMI and the expansion and aging of the global population. Despite an improved understanding of the pathogenesis of obesity and its associated health complications, BMI has not shown a decreasing trend in recent decades, instead continuing to increase worldwide.^20^, ^21^, ^22^, ^23^

Studies show that the age‐standardized incidence of EC varies by region and is decreasing in countries with ESCC as the predominant histological subtype, whereas it is increasing in regions with ECA as the predominant subtype. In 2012, nearly 30% (11 300 cases) of EAC cases worldwide were caused by excess weight.^24^, ^25^ The risk of EAC increases by 48% for every 5‐unit increase in BMI, and the association is even stronger in nonsmokers, with a 62% increase in risk for every 5‐unit increase in BMI. However, a negative association between overweight and ESCC has been reported.^26^ This negative association may be due to reverse causality or residual confounding or effect modification of smoking,^27^, ^28^, ^29^ as smokers tend to have lower BMI than nonsmokers.^30^, ^31^, ^32^, ^33^

The overall burden of EC due to high BMI is higher in areas with a high SDI, with nearly four‐fifths of deaths occurring in areas with high and middle SDI, in which the rate of increase has slowed in recent years. Although the baseline burden of EC associated with high BMI was relatively low in areas with a low SDI, the number and rate of deaths and DALYs showed a clear increasing trend. Differences in SDI may also contribute to changes in the burden of EC associated with high BMI at the national level, which parallels the pattern of distribution and change in high BMI, with national wealth being the most obvious systemic driver.^7^, ^34^ The increase in the prevalence of high BMI started in the 1970s in affluent Western nations, which experienced the greatest absolute growth.^21^ Conversely, low‐ and middle‐income countries witnessed a delayed onset of high BMI prevalence.^21^ This phenomenon is primarily attributed to the adoption of a “Western lifestyle,” characterized by the consumption of copious quantities of nutrient‐deficient, high‐calorie foods, coupled with decreased levels of physical activity.^35^ However, there are exceptions. For example, obesity rates are low in high‐income Asia‐Pacific countries, which led to a decrease in the ASMR and ASDR for high BMI‐associated EC. This may be due to adherence to a traditional diet, which favors the consumption of fewer calories, and an active transportation system that often includes walking as part of daily activities.^21^, ^36^

In the GBD 2019 study, the impact of high BMI on the burden of EC was assessed by evaluating mortality and DALYs, which account for both incidence and case fatality rates.^37^ The latter can be influenced by various factors, such as timely diagnosis, treatment accessibility, tumor biology, and the effectiveness of cancer management. Therefore, the observed surge in EC mortality rates linked to high BMI in underdeveloped nations may be attributed not only to the escalating prevalence of high BMI, but also to an inadequate healthcare infrastructure.^38^

High BMI is a modifiable risk factor for EC. Implementing policies and actions aimed at preventing and controlling excess bodyweight may be a more feasible approach to reducing the incidence of EC than addressing the effect of genetic predisposition and aging. This aligns with the World Health Organization's objective of addressing the escalating global burden of noncommunicable diseases.^39^ Although achieving this goal seems unlikely, evidence‐based and economical strategies involving a healthy diet and exercise have been tried in some countries, and their viability and feasibility have been demonstrated.^40^, ^41^ Observational studies provide compelling evidence that weight loss and exercise can mitigate disease risk and enhance survival in various cancers.^42^ Furthermore, long‐term follow‐up studies on bariatric or metabolic surgery for morbid obesity have indirectly demonstrated that weight loss can lower cancer risk in the obese population.^43^, ^44^, ^45^ Nevertheless, the implementation of weight‐loss interventions in the cancer population has yet to become standard clinical practice.

The present results provide an estimation of the contribution of high BMI to the incidence of EC and serve as a valuable point of reference for designing strategies for mitigating this issue and evaluating their efficacy over time. However, it is important to acknowledge the limitations of this investigation. First, despite the implementation of various measures by GBD 2019 to improve the quality and comparability of the data, the presence of bias cannot be entirely eliminated, potentially undermining the dependability and precision of our findings. Second, although BMI is widely used as a measurement tool, individuals with identical BMI values exhibit distinct patterns of obesity and body fat percentages, which can be attributed to variables such as age, gender, and ethnicity. Third, despite differences in the epidemiological features of the two most prevalent histological subtypes of EC, data pertaining to these subtypes have not been isolated in GBD 2019. Finally, EC is a multifaceted process, and additional factors may interact with elevated BMI to impact the likelihood of EC onset.

In conclusion, the worldwide incidence of EC linked to high BMI is on the rise, indicating a significant strain on global healthcare systems. High BMI rates are prevalent in high‐income nations and continue to escalate in most demographic groups, particularly in underdeveloped countries with inadequate cancer management. Despite compelling evidence supporting the association between exercise, weight loss, and reduced disease risk, as well as improved survival in various cancer types, effective weight loss interventions in cancer populations have yet to become routine clinical practice. Hence, a collaborative effort among governments, stakeholders, civil societies, healthcare providers, and individuals should be undertaken to increase awareness of the detrimental impact of elevated BMI, encourage the adoption of healthy dietary habits and physical exercise, and enhance healthcare services for the obese population, with the ultimate goal of mitigating the burden of EC attributable to high BMI.

## AUTHOR CONTRIBUTIONS

Zhiming Chen and Xingxing Zhang: project administration and drafting; Jiayang Fan and Jianxue Zhai: data analysis and validation; Yikuan Cai and Tianlan Ye: data collection and collation; Zhizhi Wang and Kaican Cai: supervision and drafting and editing. All authors read and approved the final manuscript.

## FUNDING INFORMATION

This project was supported by the Dean Research Funding of Nanfang Hospital, Southern Medical University, China (2022A028) and Special Foundation of President of Nanfang Hospital (2021C025).

## CONFLICT OF INTEREST STATEMENT

The authors declare no competing interests.

## Supporting information


File S1.

File S2.
**TABLE S1.** Top 10 countries or territories with the highest number of esophageal cancer deaths attributable to high body‐mass index in 2019.
**TABLE S2.** Top 10 countries or territories with the highest number of esophageal cancer DALYs related to high body‐mass index in 2019.
**TABLE S3.** Top 10 countries or territories with the highest esophageal cancer ASMR (per 100 000) attributable to high body‐mass index in 2019.
**TABLE S4.** Top 10 countries or territories with the highest esophageal cancer ASDR (per 100 000) attributable to high body‐mass index in 2019.
**TABLE S5.** Top 10 countries or territories with the highest or lowest EAPC in the ASMR (per 100 000) attributable to high body‐mass index, 1990–2019.
**TABLE S6.** Top 10 countries or territories with the highest or lowest EAPC in the ASDR (per 100 000) attributable to high body‐mass index, 1990–2019.

File S3.
**TABLE S7.** Percentage change across countries of the fraction of all esophageal cancer deaths attributable to high body‐mass index (95% CI).

File S4.
**TABLE S8.** Percentage change across countries of the fraction of all esophageal cancer DALYs attributable to high body‐mass index (95% CI).

File S5.

File S6.

File S7.

File S8.

File S9.

File S10.

## Data Availability

The Global Burden of Disease (GBD) study estimates supporting the conclusions of this article are available from the Institute for Health Metrics and Evaluation (IHME) GBD Results Tool | Global Health Data Exchange, https://vizhub.healthdata.org/gbd-results/. Human Data on the Human Development Index (HDI) was obtained from the United Nations Development Program (https://hdr.undp.org/data-center).
