# Supplementary figures and images for: Global burden of esophageal cancer attributable to high BMI in 204 countries and territories: 1990–2019
Source: Thorac Cancer. 2024 Feb 5;15(9):681–92. doi: 10.1111/1759-7714.15239 (PMC10961222; doi:10.1111/1759-7714.15239)

A

## Deaths

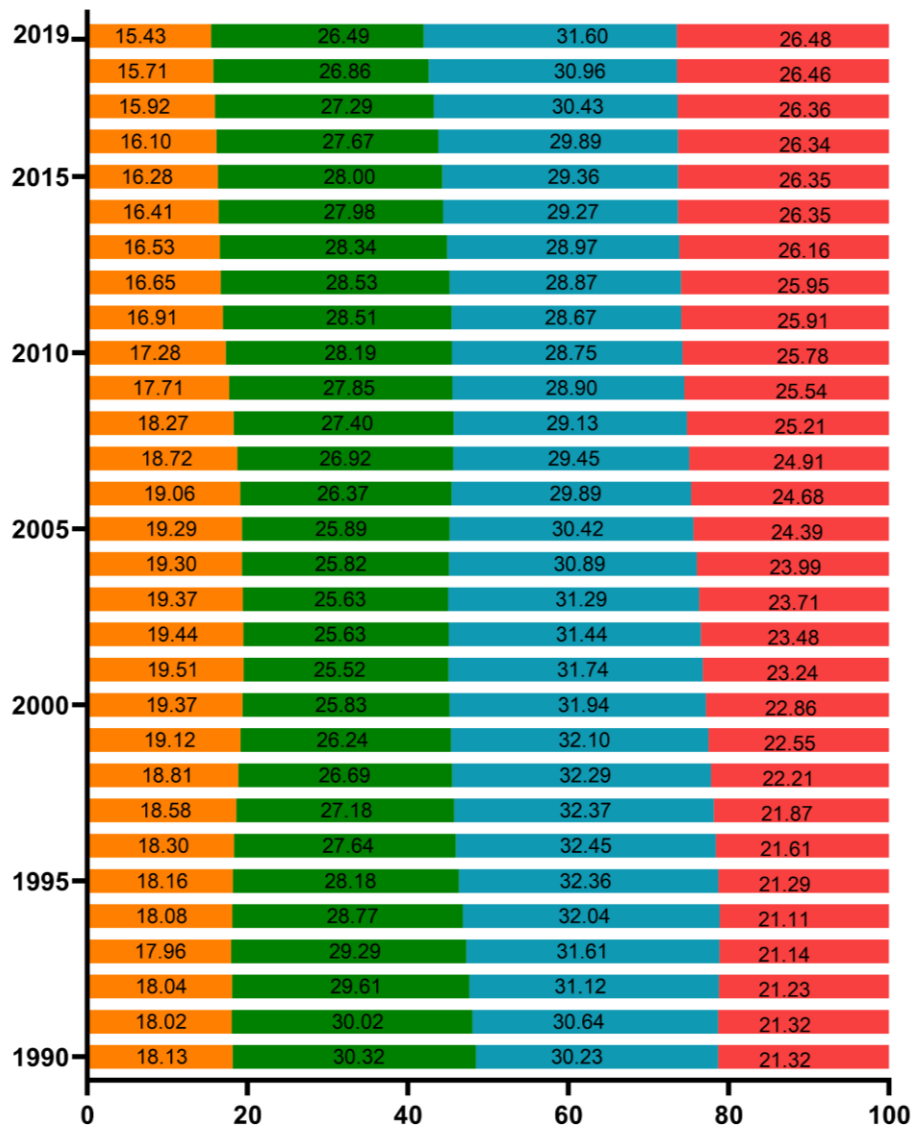

B

## DALYs (Disability-Adjusted Life Years)

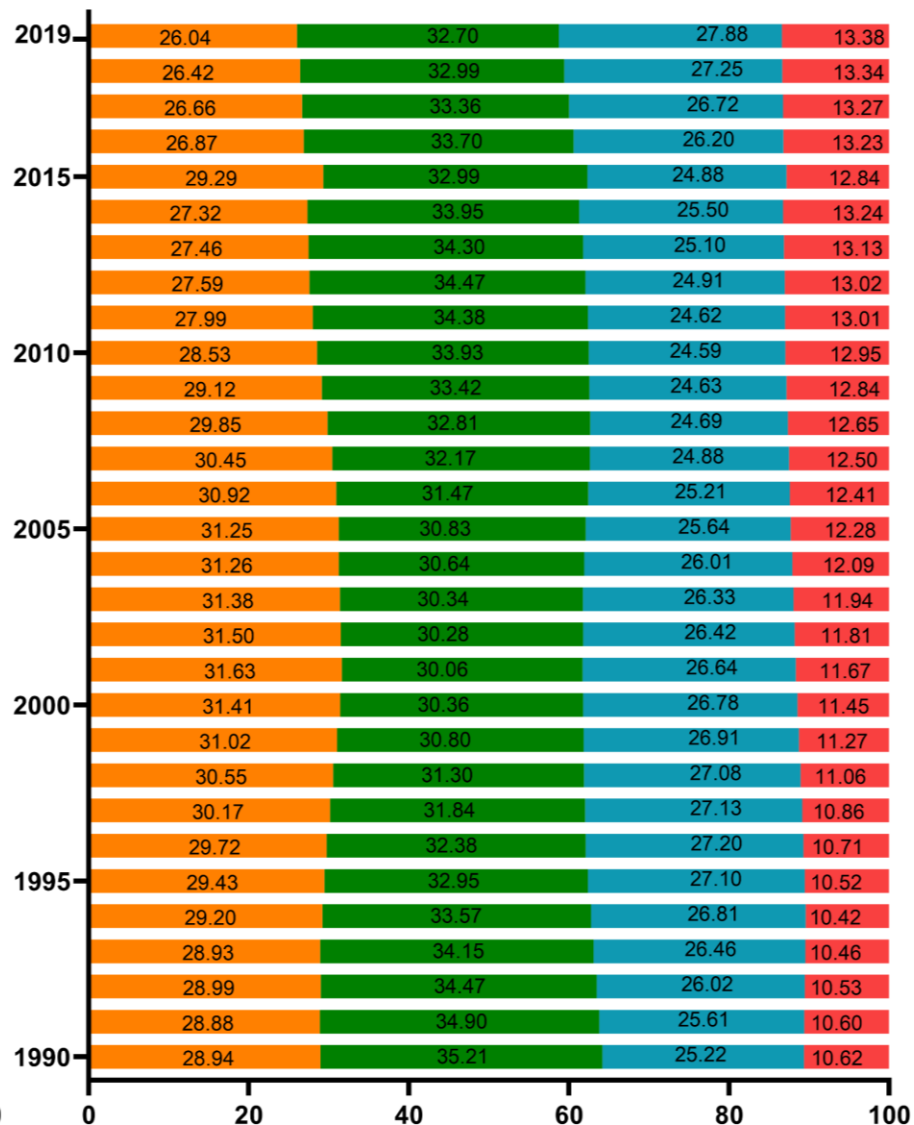

age

20-54

55-64

65-74

75+

Supplement: Supplementary file 1 — File S1. [file TCA-15-681-s007.pdf]

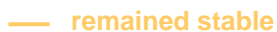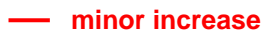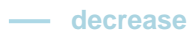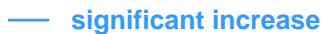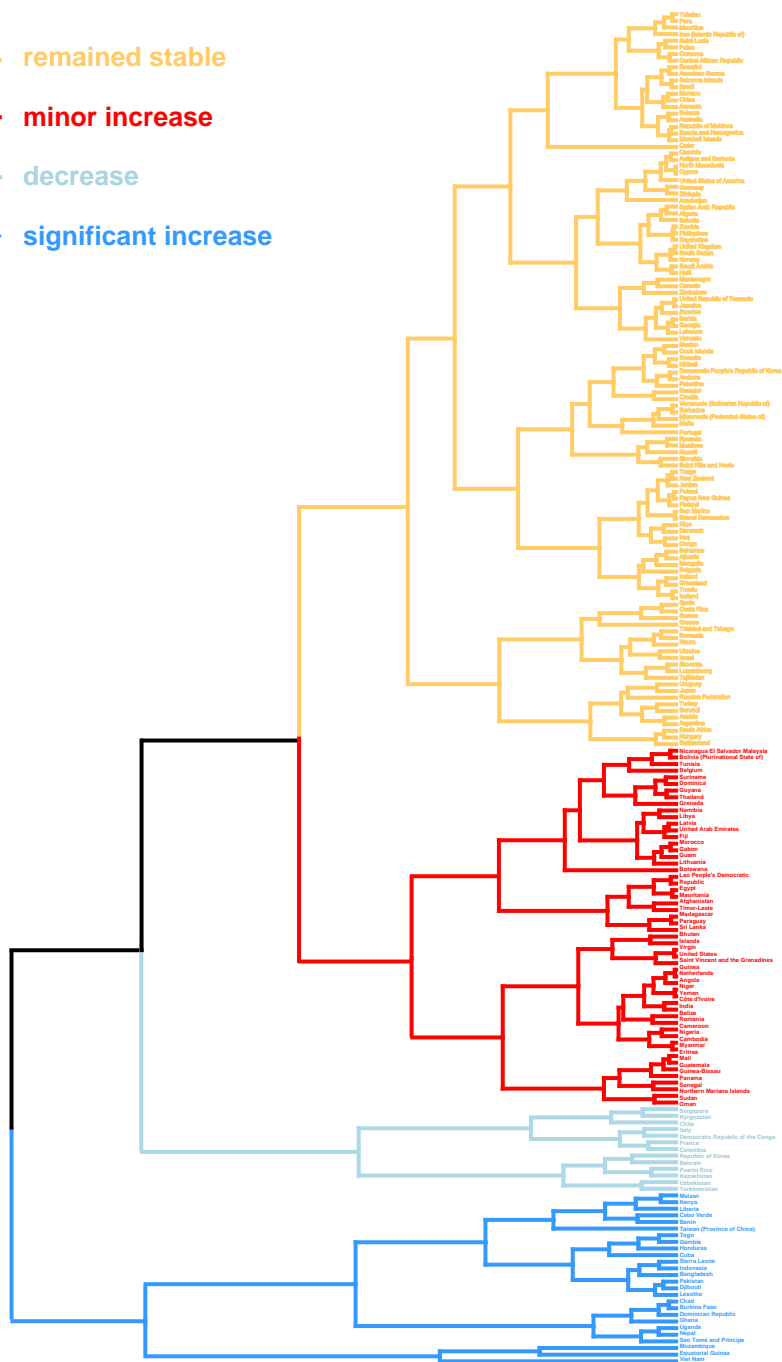

Supplement: Supplementary file 5 — File S5. [file TCA-15-681-s003.pdf]

R = -0.015

P = 0.834

Age-standardized Deaths rate (per 100000 populations)

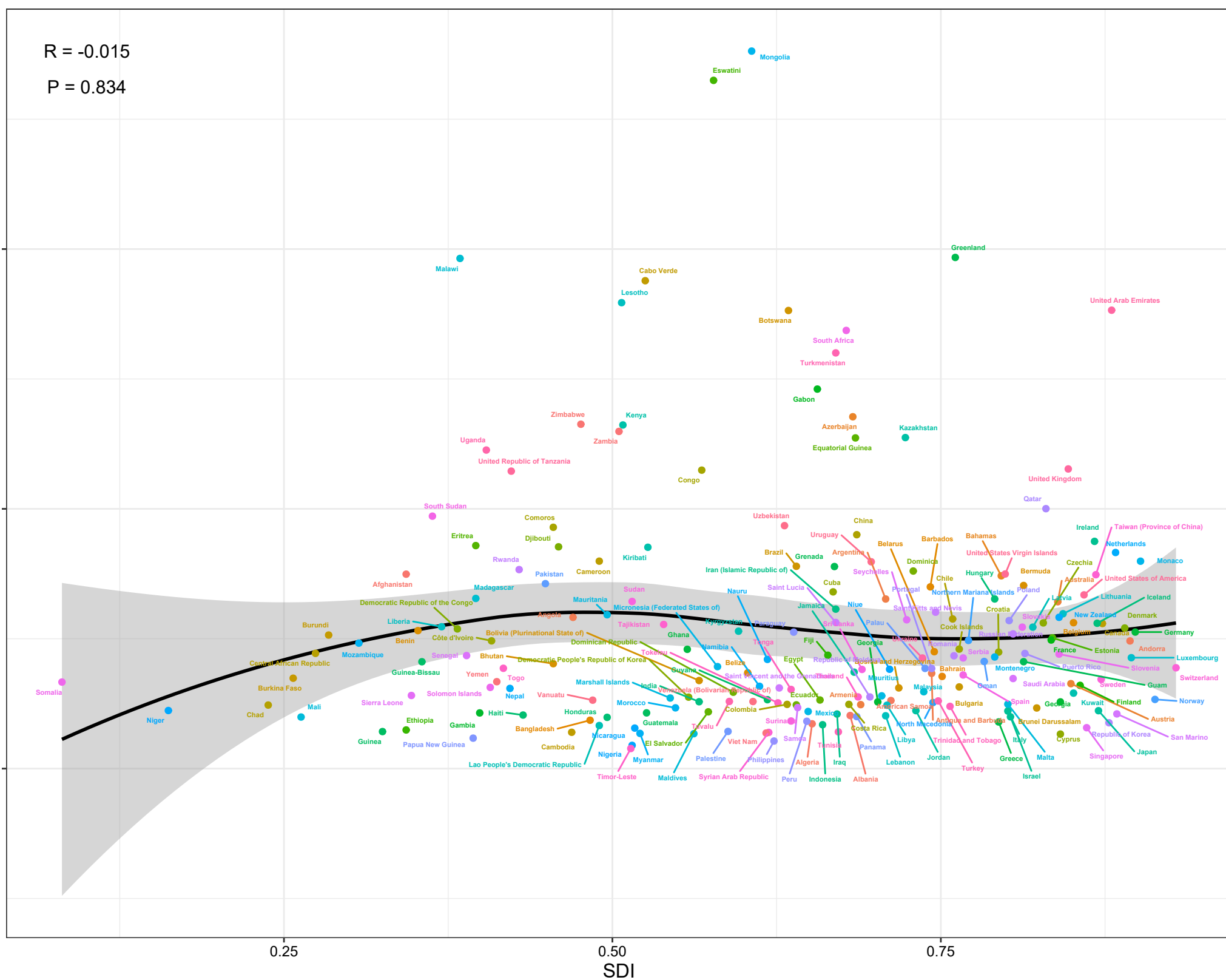

Supplement: Supplementary file 6 — File S6. [file TCA-15-681-s001.pdf]

R = -0.047

P = 0.504

Age-standardized DALYs rate (per 100000 populations)

100

50

0

0.25

0.50  
SDI

0.75

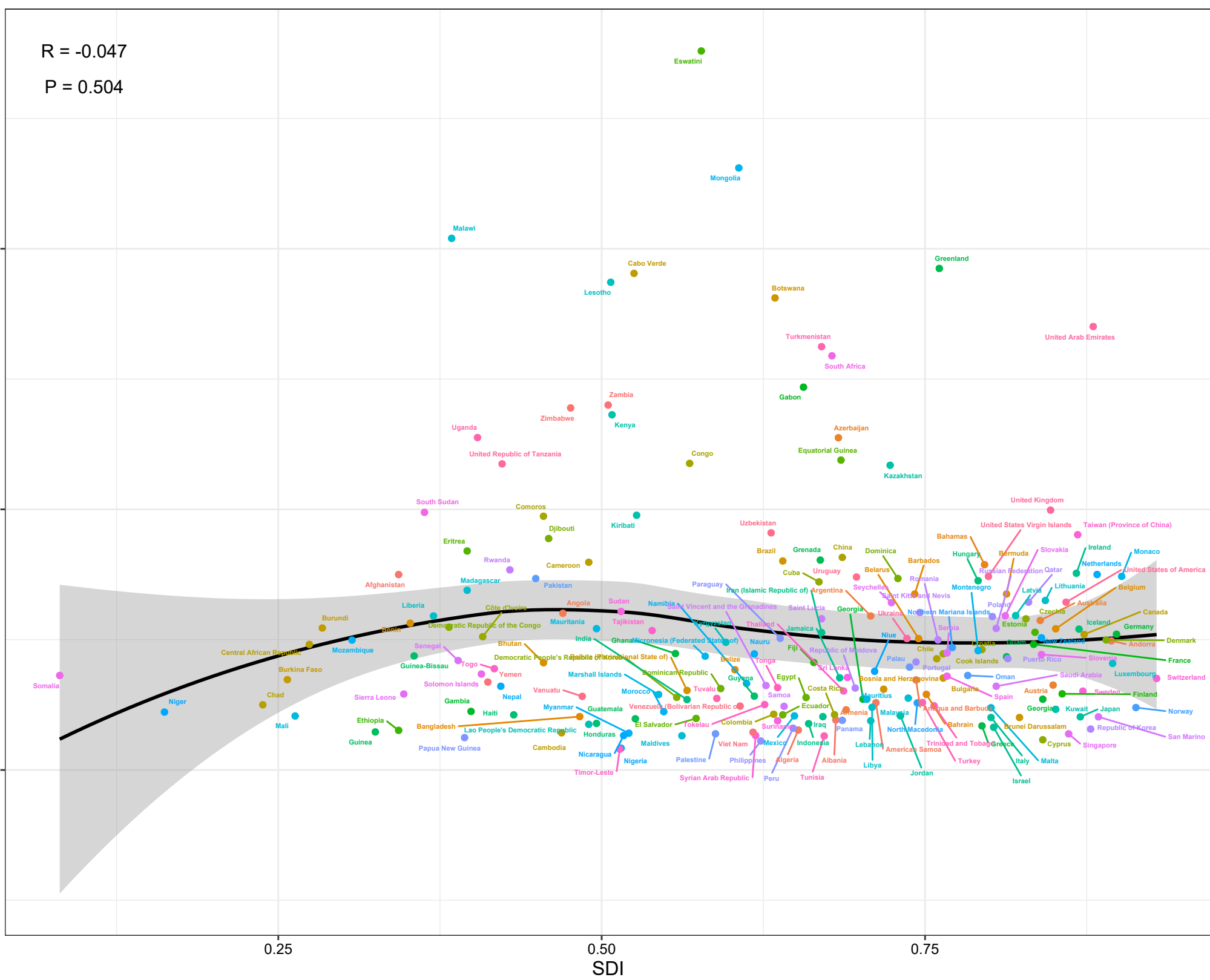

Supplement: Supplementary file 7 — File S7. [file TCA-15-681-s005.pdf]

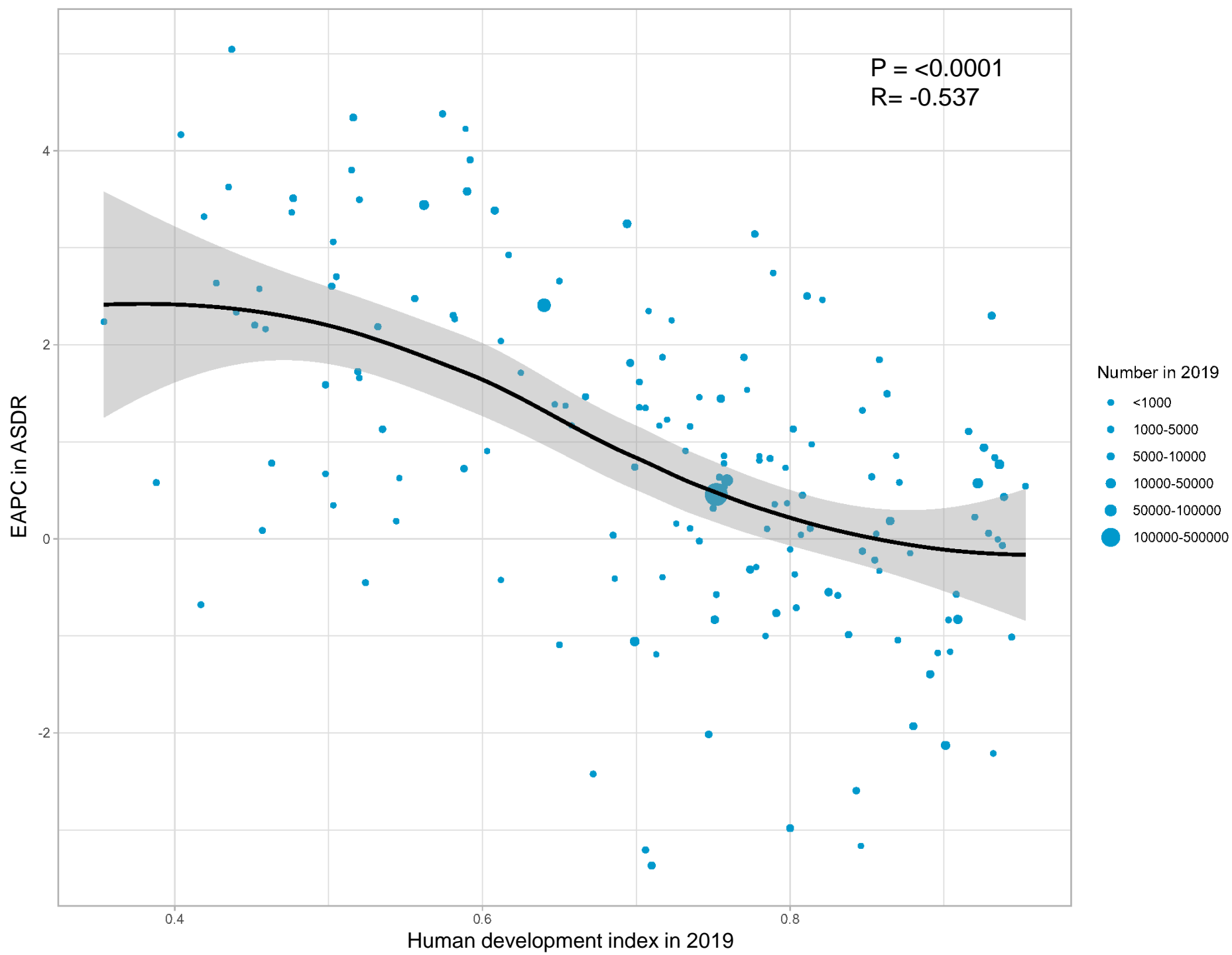

Supplement: Supplementary file 8 — File S8. [file TCA-15-681-s010.pdf]

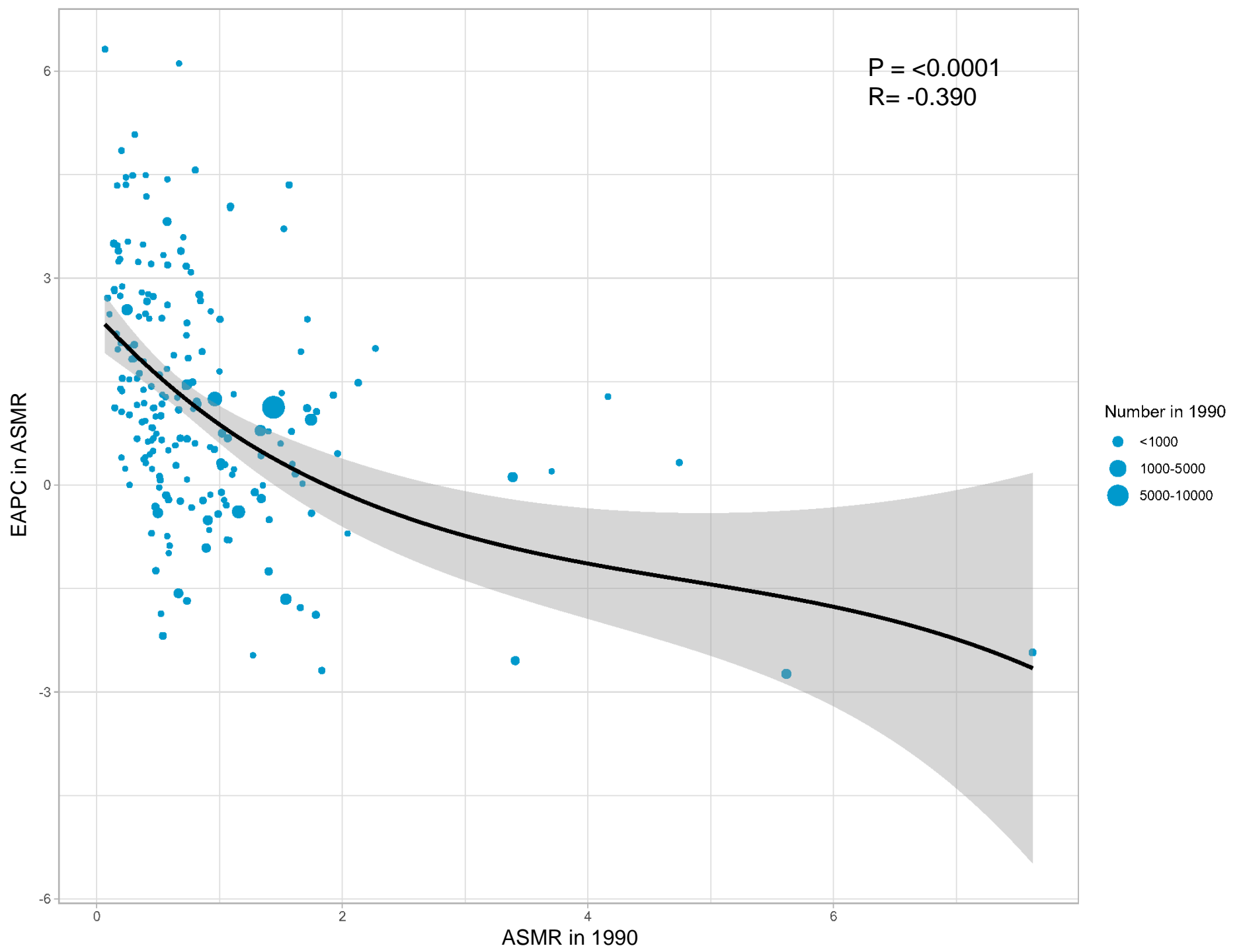

Supplement: Supplementary file 9 — File S9. [file TCA-15-681-s008.pdf]

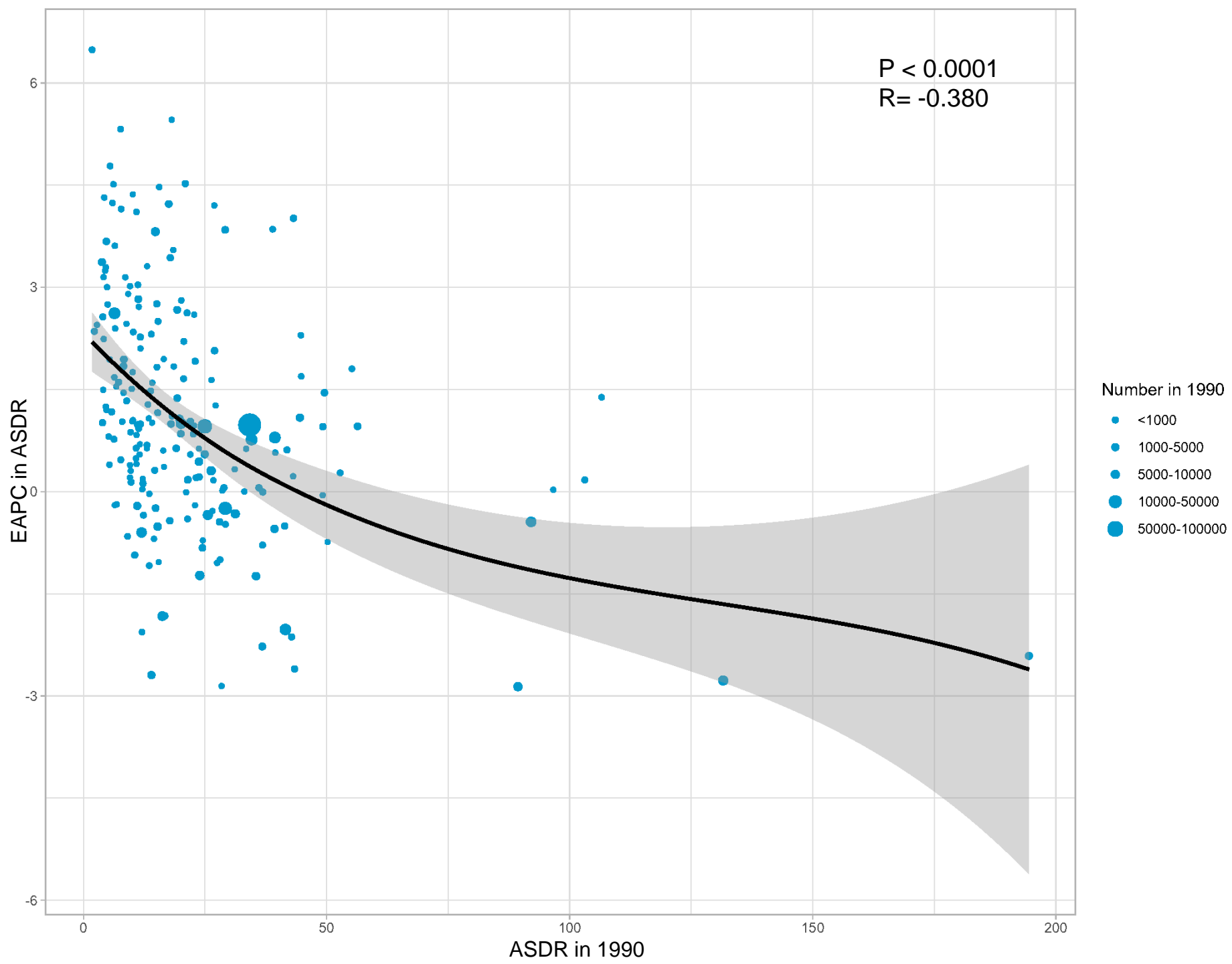

Supplement: Supplementary file 10 — File S10. [file TCA-15-681-s009.pdf]
